# Psychoradiology: a new era for neuropsychiatric imaging

**DOI:** 10.1093/psyrad/kkaa001

**Published:** 2021-03-18

**Authors:** Qiyong Gong, Keith M Kendrick, Lin Lu

**Affiliations:** Huaxi MR Research Center (HMRRC), Department of Radiology, West China Hospital of Sichuan University, Chengdu, Sichuan Province, China; Research Unit of Psychoradiology, Chinese Academy of Medical Sciences, Chengdu, Sichuan Province, China; Neurotherapy and Social, Cognitive and Affective Neuroscience Center, University of Electronic Science and Technology, Chengdu, Sichuan Province, China; Institute of Mental Health/Peking University Sixth Hospital, Peking University, China

We are very pleased to introduce the first issue of *Psychoradiology*, an open-access journal that publishes original research articles, reviews, case reports, editorials, commentaries, correspondence, and perspectives. The journal will publish high-quality and interdisciplinary manuscripts that span the genetic and molecular basis of psychiatric disorders to social and cultural factors that influence the onset, progress, and behavioral symptoms of these illnesses. In particular, the journal aims to showcase research that promotes the integration of medical imaging biomarkers into clinical diagnosis, prognostic prediction, and treatment development, as well as the efficacy of brain stimulation, pharmacological, and behavioral training interventions. We are excited about this new journal, and believe that it can contribute greatly to dissemination of important discoveries and new approaches in basic and clinical science in the fields of radiology, psychology, psychiatry, neurology, and neuroscience, as well as medical imaging, interventional medicine, computation, brain-machine interfaces, and artificial intelligence (AI).

A field once limited to observation, description, supportive management, and clinical investigation in psychiatric disorders has undergone an exciting evolution that now incorporates quantitative exploration of neural networks and mechanisms, auxiliary diagnosis by radiomics, machine learning based prediction of prognosis, and genetic, behavioral, and imaging biomarker-guided development of treatments thanks particularly to advances in neuroscience, imaging technology with Magnetic Resonance Imaging (MRI), and data-driven AI approaches. Psychoradiology, i.e. clinical psychiatric imaging, is a rapidly emerging subfield of radiology that utilizes advanced neuroimaging approaches to improve differential diagnosis and individualized patient care for common psychiatric illnesses. This new journal, *Psychoradiology*, aims to bridge the gap between neuroscientists and clinicians by providing a platform for publishing research using brain imaging, brain stimulation, and psychological approaches that have translational relevance for the diagnosis, prognosis, prevention, and/or treatment of psychiatric and neurological disorders.

MRI has been the mainstay and a powerful tool for psychoradiology, in conjunction with the application of AI. These advancements are not only providing an insightful understanding of underlying pathological mechanisms but also expediting the translation of psychoradiological discoveries into clinical care for patients with neuropsychiatric disorders. The possibility of multimodal MRI allowing for assessment of brain tissue at structural, functional, and molecular levels has helped the translation of psychoradiology into clinical practice. Both qualitative and quantitative imaging markers of mental disorders have been identified and are starting to impact clinical workflow. Other medical imaging modalities including positron emission tomography, electroencephalogram, magnetoencephalogram, functional near-infrared spectroscopy, etc. have also helped to deepen our understanding of the mechanisms of psychiatric disorders, providing objective detection and early diagnosis, and enabling prognosis and early treatment. The status of psychoradiology in clinical practice, and its potential to identify and subtype patients and measure psychopharmaceutical effects, together with future challenges will be discussed in the first issue of *Psychoradiology*.

As editors, one of our aspirations for *Psychoradiology* is that it will benefit from the development and maturation over the last four decades of sophistication in experimental design, data acquisition, analysis, and interpretation in medical imaging-based fundamental research in psychiatric disorders. The challenges are also multiplied as comparisons are made across different populations and new methods for data analysis are developed and advanced in leaps and bounds. Critically, however, clinically relevant results are of paramount importance, since simple group-wise differences, expressed in terms of impaired brain structure or function, offer limited explanatory value for advancing our understanding of psychiatric illnesses in terms of personalized treatment. At *Psychoradiology*, we seek hypothesis-based studies in which imaging measures are used in realistic and creative ways to shed new light on our understanding of problems experienced by patients with clinical neurological and psychiatric disorders. We anticipate that such contributions will help establish a platform to optimize the impact of psychoradiological findings and offer the opportunity for scientists and clinicians to shape and direct this emergent field through the publication of timely reviews, commentary, and perspective articles.

For sharing scientific and academic advances and updates throughout the world, *Psychoradiology* acknowledges that publications will have greater visibility, wider circulation, and easier access for a wider readership through its open-access publishing policy, and this will be especially important for economically disadvantaged regions of the world. Prospective authors are welcome to submit manuscripts at https://mc.manuscriptcentral.com/psyrad and please feel free to contact us if there are questions about a submission.

